# Voltage-Gated Na^+^ Channel Isoforms and Their mRNA Expression Levels and Protein Abundance in Three Electric Organs and the Skeletal Muscle of the Electric Eel *Electrophorus electricus*

**DOI:** 10.1371/journal.pone.0167589

**Published:** 2016-12-01

**Authors:** Biyun Ching, Jia M. Woo, Kum C. Hiong, Mel V. Boo, Wai P. Wong, Shit F. Chew, Yuen K. Ip

**Affiliations:** 1 Department of Biological Sciences, National University of Singapore, Kent Ridge, Republic of Singapore; 2 The Tropical Marine Science Institute, National University of Singapore, Kent Ridge, Republic of Singapore; 3 Natural Sciences and Science Education, National Institute of Education, Nanyang Technological University, Republic of Singapore; Vanderbilt University Medical Center, UNITED STATES

## Abstract

This study aimed to obtain the coding cDNA sequences of *voltage-gated Na*^*+*^ *channel* (*scn*) *α*-subunit (*scna*) and *β*-subunit (*scnb*) isoforms from, and to quantify their transcript levels in, the main electric organ (EO), Hunter’s EO, Sach’s EO and the skeletal muscle (SM) of the electric eel, *Electrophorus electricus*, which can generate both high and low voltage electric organ discharges (EODs). The full coding sequences of two *scna* (*scn4aa* and *scn4ab*) and three *scnb* (*scn1b*, *scn2b* and *scn4b*) were identified for the first time (except *scn4aa*) in *E*. *electricus*. In adult fish, the *scn4aa* transcript level was the highest in the main EO and the lowest in the Sach’s EO, indicating that it might play an important role in generating high voltage EODs. For *scn4ab*/Scn4ab, the transcript and protein levels were unexpectedly high in the EOs, with expression levels in the main EO and the Hunter’s EO comparable to those of *scn4aa*. As the key domains affecting the properties of the channel were mostly conserved between Scn4aa and Scn4ab, Scn4ab might play a role in electrogenesis. Concerning *scnb*, the transcript level of *scn4b* was much higher than those of *scn1b* and *scn2b* in the EOs and the SM. While the transcript level of *scn4b* was the highest in the main EO, protein abundance of Scn4b was the highest in the SM. Taken together, it is unlikely that Scna could function independently to generate EODs in the EOs as previously suggested. It is probable that different combinations of Scn4aa/Scn4ab and various Scnb isoforms in the three EOs account for the differences in EODs produced in *E*. *electricus*. In general, the transcript levels of various *scn* isoforms in the EOs and the SM were much higher in adult than in juvenile, and the three EOs of the juvenile fish could be functionally indistinct.

## Introduction

Some fishes have acquired the ability to generate and/or sense electricity [1]. Electric fishes can generate strong electric organ discharges (EODs) for predation and defense or weak EODs for communication and sensing of the surroundings [2]. The electric eel, *Electrophorus electricus*, is a strongly electric fish belonging to the Order Gymnotiformes and the Family Gymnotida. Its natural geographical range includes the northeastern South America, covering parts of the Amazon, Guyanas and Orinoco Rivers [3].

Unlike other electric fishes, *E*. *electricus* has three electric organs (EOs), the main EO, the Hunter’s EO and the Sach’s EO. The main EO of *E*. *electricus* produces high voltages EODs up to 600 V at a frequency of several hundred Hz, while the Sach’s EO produces low voltage EODs of about 10 V at a frequency of up to 25 Hz [4]. The Hunter’s EO can produce both high and low voltage EODs, at the anterior and posterior regions of the organ, respectively [2,5,6]. The ability of a mature *E*. *electricus* to generate a burst of EOD peaking at 600 V with a current of 2 A within one second is the highest among animals [7]. The low-frequency, low-voltage EODs generated by the Sach’s EO and the posterior one-third of the Hunter’s EO are mainly used for navigation and communication purposes [8]. *Electrophorus electricus* hunts its prey using high-frequency voltage pulses which are targeted to motor neurons, resulting in the induction of uncontrolled muscle seizures [9]. It can also generate a doublet of electric discharge during hunting to cause muscle twitching in stationary or hidden prey, thus revealing the prey’s location [9].

EOs comprise electrocytes which have myogenic origins; newly-forming electrocytes derived from embryonic myoblasts are long, multinucleated and ribbon-like [10], while mature electrocytes have a more flattened disc-like appearance. Each electrocyte has a rostral non-innervated membrane, and a posterior membrane innervated by electromotor neurons [11,12]. Nicotinic acetylcholine receptor, acetylcholinesterase, and voltage-gated Na^+^ channel (Scn) are localized to the innervated membrane, but absent from the non-innervated membrane, thereby resulting in a polarization of chemical and electrical excitability among the two different surfaces of the electrocyte [11,13–16]. When the electrocytes are stimulated, Scn on the innervated membrane open to allow an influx of Na^+^ to depolarize the innervated membrane of the electrocyte [15,17]. This generates a transcellular electrical potential, and the summation of potentials across a series of electrocytes along the EO results in the generation of a large EOD. Peak currents generated by the main EO are much higher than those generated by the Sach’s EO, attributable mainly to the more densely-packed electrocytes with higher innervated membrane Scn densities in the former as compared with the latter [11,18].

A *scn* α-subunit (*scna*) was first cloned and sequenced from *E*. *electricus* (*Electrophorus electricus* sodium channel mRNA, complete cds; Accession: M22252.1) [19–22]. It was proposed originally that this Scna could function by itself without the association with any Scn β-subunit (Scnb) [23]. However, for SCN in the mammalian brain and muscle tissue, SCNA is known to be associated with one or two SCNB [24,25]. In mammals, there are 10 different isoforms of SCNA expressed in various tissues [26]. SCNA take part fundamentally in the depolarization of excitable cells, leading to action potential propagation in neuronal cells and triggering contractions in skeletal muscle (SM) [27]. There are four types of SCNB; SCN1B and SCN3B are associated non-covalently with SCNA, but SCNA forms disulphide bonds with SCN2B and SCN4B [26]. SCNB isoforms are important in modulating the SCNA functions; they may change the biophysical properties of the channel, such as increasing the membrane capacitance and current amplitude, and modulating its gating [28]. Therefore, this study was undertaken to obtain the coding cDNA sequences of *scna* and *scnb* isoforms from the main EO, the Hunter’s EO, the Sach’s EO and the SM of *E*. *electricus*, and to identify the isoform types based on their deduced amino acid sequences. We have successfully obtained the full coding sequences of *scn4aa* and *scn4ab* from *E*. *electricus*, and additionally discovered the expression of several *scnb* isoforms in the EOs and the SM. To test the hypothesis that certain *scna* and/or *scnb* isoforms could be EO- or SM-predominant, the transcript levels of *scna* and *scnb* isoforms in the EOs and the SM were determined by quantitative real-time polymerase chain reaction (qPCR). It was hoped that results obtained would elucidate the possible relationships between certain *scn* subunit isoforms and the ability to produce strong or weak electric discharges among the three EOs. Furthermore, based on the deduced amino acid sequences, custom-made anti-Scn4ab and anti-Scn4b antibodies were raised and western blotting performed in order to compare the protein abundances of certain Scn isoforms among the three EOs and the SM. As immunoblotting and immunohistochemical work on Scn4aa had been performed previously in *E*. *electricus* [29,30], Western blotting in this study focused on Scn4ab. As for Scnb, the focus was on Scn4b because it exhibited the highest mRNA expression level compared with other Scnb isoforms. Efforts were also made to compare the mRNA expression levels and protein abundances of *scna*/Scna and *scnb*/Scnb isoforms between adult and juvenile *E*. *electricus*, and to test the hypothesis that differences existed at the molecular level between EOs of adults and EOs of juveniles.

### A note on gene and protein nomenclature

Two different types of abbreviations of genes/proteins have been adopted in this report, as the standard abbreviations of genes/proteins of fishes (http://zfin.org/cgi-bin/webdriver?MIval=aa-ZDB_home.apg) are different from those of human/non-human primates (http://www.genenames.org). For fishes, gene symbols are italicized, all in lower case, while protein designations are the same as the gene symbol and not italicized with the first letter in upper case.

## Materials and Methods

### Fish

*Electrophorus electricus* (juvenile: 200–300 g, *N* = 4; adult 1800–2300 g, *N* = 4) were imported from South America through a local fish farm in Singapore. No attempt was made to separate the sexes. Fish were maintained in dechlorinated tap water at 25°C and acclimated to laboratory conditions for one week. Fish were killed with an overdose of neutralized MS-222 (0.2%) followed with a strong blow to the head. Tissue samples of the three EOs and the SM were excised, frozen in liquid nitrogen and stored at -80°C until analyses.

### Ethics statement

Approval to undertake this study was granted by the Institutional Animal Care and Use Committee of the National University of Singapore (IACUC 142/12).

### Total RNA extraction and cDNA synthesis

Total RNA was extracted from the tissue sample using Tri Reagent^™^ (Sigma-Aldrich Co., St. Louis, MO, USA), and further purified using the RNeasy Plus Mini Kit (Qiagen GmbH, Hilden, Germany). Following isolation, RNA was quantified spectrophotometrically using Shimadzu BioSpec-nano (Shimadzu, Tokyo, Japan). RNA integrity was verified electrophoretically and the RNA was stored at -80°C. First strand cDNA was synthesized from 4 μg of total RNA using oligo(dT)_18_ primer and the RevertAid^™^ first strand cDNA synthesis kit (Thermo Fisher Scientific Inc.).

### Polymerase chain reaction (PCR)

Partial *scn4aa*, *scn4ab*, *scn1b*, *scn2b and scn4b* sequences were obtained using PCR primers (S1 Table). PCR was performed in a Biorad Peltier thermal cycler (Biorad Laboratories, Hercules, CA, USA) using Dreamtaq polymerase (Thermo Fisher Scientific Inc.). The cycling conditions were 95°C for 3 min, followed by 40 cycles of 95°C for 30 s, 60°C for 30 s, 72°C for 2 min and a final extension of 72°C for 10 min. PCR products were separated by electrophoresis in 1% agarose gel. Bands of predicted molecular masses were excised and purified using FavorPrep^™^ Gel Purification Mini Kit (Favorgen Biotech Corp., Ping-Tung, Taiwan) according to manufacturer’s protocol. Purified PCR products were subjected to cycle sequencing using BigDye^®^ Terminator v3.1 Cycle Sequencing Kit (Life Technologies Corporation, Carlsbad, California) and sequenced using the 3130XL Genetic Analyzer (Life Technologies Corporation).

### Rapid amplification of cDNA ends (RACE)-PCR

Total RNA (1 μg) isolated from the tissue sample was reverse transcribed into 5’-RACE-Ready cDNA and 3’RACE-Ready cDNA using SMARTer^™^ RACE cDNA Amplification kit (Clontech Laboratories, Mountain View, CA, USA). RACE-PCR was performed using the Advantage^®^ 2 PCR kit (Clontech Laboratories) to generate the 5’ and 3’ cDNA fragments. The cycling conditions comprised 30 cycles of 94°C for 30 s, 65°C for 30 s, and 72°C for 4 min. RACE-PCR products were separated using gel electrophoresis, purified and sequenced. Multiple sequencing was performed in both directions to obtain the full-length cDNA. Sequence assembly and analysis were performed using Bioedit v7.1.3 [31].

### Deduced amino acid sequences, classification tables and molecular characterization

The Scn amino acid sequences were translated from the respective *scn* nucleotide sequences using ExPASy Proteomic server (http://web.expasy.org/translate/). Amino acid sequences of SCNA/Scna and SCNB/Scnb from other animals obtained from Genbank or UniProtKB/TrEMBL (S2 Table) were aligned using ClustalW and percentage similarity calculated using BioEdit [31]. The transmembrane domains were predicted using MEMSAT3 and MEMSAT-SVM provided by PSIPRED protein structure prediction server (http://bioinf.cs.ucl.ac.uk/psipred/) [32]. Possible phosphorylation sites were predicted using NetPhos 2.0 Server [33]. Glycosylation sites were predicted according to UniProt.

### qPCR

There are two types of quantification methods for qPCR [34]. Relative quantitation involves the comparison of the targeted gene with a reference gene, and measures only fold-change data, which does not allow the interpretation of which isoform being the predominant one expressed in a certain tissue or under a certain condition. For absolute quantification, the precise amount of the template used for the standard curve is known, and therefore results of the targeted gene can be expressed as absolute numbers of copies of transcripts. As it is essential to compare the mRNA expression levels of various *Scn* isoforms among the SM and the three EOs of *E*. *electricus*, the method of absolute quantification with reference to standard curves was adopted in this study. RNA (4 μg) from tissue samples of *E*. *electricus* were extracted as mentioned above and reverse-transcribed using random hexamer primers with RevertAid^™^ first strand cDNA synthesis kit. qPCR was performed in triplicates using a StepOnePlus^™^ Real-Time PCR System (Thermo Fisher Scientific Inc.). The mRNA expression level of *scn* isoforms were determined using specific forward and reverse qPCR primers (S3 Table).

In order to determine the absolute quantity of a specific *scn* transcript in a qPCR reaction, efforts were made to produce a pure amplicon (standard) of the region of *GS*, as defined by the qPCR primers, from the tissues of *E*. *electricus* following the method of Gerwick et al. [35]. PCR was performed with a specific pair of qPCR primers and cDNA as a template in a final volume of 25 μl with the following cycling conditions: initial denaturation 95°C for 3 min, followed by 35 cycles of 95°C for 30 s, 60°C for 30 s and 72°C for 30 s and 1 cycle of final extension of 72°C for 10 min. The PCR product was purified and cloned using pGEM^®^-T Easy vector. The presence of the insert in the recombinant clones was confirmed by sequencing. The cloned circular plasmid was quantified using a spectrophotometer.

To prepare a standard curve, the standard cDNA (template) was serially diluted (from 10^6^ to 10^2^ specific copies per 2 μl). The qPCR reactions contained 5 μl of 2x KAPA SYBR FAST qPCR Master Mix (Kapa Biosystems Inc, Wilmington, MA, USA), 0.15–0.2 μmol l^-1^ each of forward and reverse primers and various quantities of standard in a total volume of 10 μl. Cycling conditions were 95°C for 20 s (1 cycle), followed by 40 cycles of 95°C for 3 s and 60°C for 30 s. The threshold cycle (C_t_) values were collected at each elongation step. A melt curve analysis was performed after each run by increasing the temperature from 60°C to 95°C in 0.3°C increments to confirm the presence of a single product only. The PCR products obtained were also separated in a 2% agarose gel to verify the presence of a single band. A standard curve was obtained from plotting C_t_ values on the Y-axis and the natural log of concentration (copies μl^-1^) on the X-axis. The C_t_ slope, PCR efficiency, Y-intercept and correlation coefficient (*r*^*2*^) were calculated using the default setting of StepOne^™^ Software v2.1. Diluted standards were stored at -20°C. The amplification efficiencies for *scn4aa*, *scn4ab*, *scn1b*, *scn2b and scn4b* were 93.6%, 96.8%, 100.4%, 105.6% and 99.6%, respectively.

For tissue samples of *E*. *electricus* (*N* = 4), 2 μl of cDNA (equivalent to 1 or 10 ng of cDNA) was used in place of the standard in the qPCR reaction. Runs were followed by melt curve analysis by increasing from 60°C to 95°C in 0.3°C increments to confirm the presence of only a single product. Electrophoresis was also done in a 2% agarose gel to verify the presence of a single band. The quantity of *scn* transcripts in a sample was determined from the linear regression line derived from the standard curve and expressed as copy number per ng cDNA.

### Western blotting

A commercial firm (GenScript, Piscataway, NJ, USA) was engaged to raise rabbit polyclonal antibodies against amino acids 34–47 (EQQHRKSMNIEIPE) of the translated amino acid sequence of Scn4ab and amino acids 220–233 (LSGSKAETKASPKA) of the translated amino acid sequence of Scn4b of *E*. *electricus*.

The tissues sample was homogenized three times in five volumes (w/v) of ice cold buffer containing 50 mmol l^-1^ Tris HCl, (pH 7.4), 1 mmol l^-1^ EDTA, 150 mmol l^-1^ NaCl, 1 mmol l^-1^ NaF, 1 mmol l^-1^ Na_3_VO_4_, 1% NP-40, 1% sodium deoxycholate, 1 mmol l^-1^ PMSF, and 1x HALT protease inhibitor cocktail (Thermo Fisher Scientific Inc.) at 24,000 rpm for 20 s each with 10 s intervals using the Polytron PT 1300D homogenizer (Kinematica AG, Lucerne, Switzerland). The homogenate was centrifuged at 10,000 ×g for 20 min at 4°C. The protein concentration in the supernatant obtained was determined according to the method of Bradford [36] and adjusted to 1 μg μl^-1^ with Laemmli buffer [37]. The sample was heated at 70°C for 15 min, and then kept at -80°C until analysis. The protein loads were 20 μg and 5 μg for detecting Scn4ab and Scn4b respectively.

Proteins were separated by SDS-PAGE under conditions as described by Laemmli [37] using a vertical mini-slab apparatus (Bio-Rad Laboratories, Hercules, CA, USA). After SDS-PAGE, separated proteins were electrophoretically transferred onto PVDF membranes using a transfer apparatus (Bio-Rad Laboratories). After transfer, membranes were blocked with 1% skim milk in TTBS (0.05% Tween 20 in Tris-buffered saline: 20 mmol l^-1^ Tris-HCl; 500 mmol l^-1^ NaCl, pH 7.6) for 1 h before being incubated 1 h at 25°C with the anti-Scn4ab or Scn4b antibodies (1:1000 dilution). The membranes were rinsed with TTBS and incubated in goat anti-rabbit alkaline phosphatase-conjugated secondary antibody (1:10,000; Santa Cruz Biotechnology Inc.) for 1 h at room temperature. Bands were visualized by the BCIP/NBT Substrate Kit (Invitrogen Corporation, Camarillo, CA, USA). The membranes were scanned using CanonScan 4400F flatbed scanner in TIFF format at 300 dpi resolution. Densitometric quantification of band intensities were performed using ImageJ (version 1.40, NIH), calibrated with a 37 step reflection scanner scale (1” x 8” Stouffer #R3705-1C; Stouffer Graphic Arts, IN, USA).

In order to validate the specificity of the antibodies, blocking peptide competition assays were performed. The antibodies (25 μg each) were incubated with the respective immunising peptides (125 μg each) in a total volume of 200 μl for 1 h at 25°C. A parallel control was performed without the immunizing peptide. The resulting media containing the antibodies were diluted and used for Western blotting as described above.

Usually, a reference protein, e.g. β-actin or glyceraldehyde 3-phosphate dehydrogenase, is included in Western blotting to confirm that the experimental condition tested leads to changes in the abundance of the targeted protein, but not the reference protein, in the same tissue/organ as compared to the control condition. However, in this study, we aimed to compare the protein abundances of Scn subunit isoforms among 4 different tissue/organs in *E*. *electricus* whereby no change in experimental conditions, and hence no control, were involved. Preliminary studies confirmed that the protein abundances of β-actin and glyceraldehyde 3-phosphate dehydrogenase differed significantly among the three EOs and the SM of *E*. *electricus*, indicating that they were not suitable to act as reference proteins. Therefore, for valid comparisons, Western blotting results were presented as relative protein abundance of Scn4ab per 20 μg protein or relative protein abundance of Scn4b per 5 μg protein.

### Phylogenetic analysis

A phylogenetic analysis on the *scnb* isoforms was conducted following the general method of Paul et al. [38]. Coding sequences of *scn1b*, *scn2b* and *scn4b* from *E*. *electricus* were aligned with selected sequences from various fish species (S4 Table) using ClustalW (Bioedit) and the ends were trimmed (S1 File). The best-fitting evolutionary model under the Akaike Information Criterion (AIC) was determined to be GTR (general time-reversible model) using ModelGenerator v0.85 [39]. A Bayesian analysis was conducted using MrBayes v3.2.6 [40]. The GTR model was implemented and two independent runs were processed for 3 million generations. The average standard deviation of split frequencies reached 0.006941. The consensus tree was obtained with the first 25% of runs discarded as burnin. The tree was rooted against *Latimeria chalumnae scn4A*. A maximum likelihood (ML) analysis was also run using RaxML v8.2.5 (AVX) [41] with 1000 bootstraps. Trees were determined to have converged after 750 replicates by the bootstrap convergence criterion.

### Statistical analysis

Results were presented as means ± standard errors of means (S.E.M.). Statistical analyses were performed using SPSS version 21 (IBM Corporation, Armonk, NY, USA). Square root transformation was applied to non-normally distributed datasets. Homogeneity of variance was checked using Levene’s Test. One-way analysis of variance (ANOVA), followed by multiple comparisons of means by the Tukey or Dunnett T3 post-hoc test, were used in the evaluation of the differences between means where applicable. Differences were regarded as statistically significant at P<0.05.

## Results

### Nucleotide and amino acid sequences of *scn*/Scn subunits and isoforms

The complete coding cDNA sequences of *scn4aa* (Accession: KX575860), *scn4ab* (Accession: KX575856), *scn1b* (Accession: KX575857), *scn2b* (Accession: KX575858), and *scn4b* (Accession: KX575859) were obtained from the EOs and the SM of *E*. *electricus*. The complete coding sequence obtained for *scn4aa* consisted of 5463 bp coding for 1820 amino acids with a calculated molecular mass of 208.3 kDa. The complete coding sequence of *scn4ab* consisted of 5178 bp coding for 1725 amino acids with an estimated molecular mass of 196.4 kDa. For *scn1b*, the complete coding sequence was 630 bp, coding for 209 amino acids with an estimated molecular mass of 23.6 kDa. The *scn2b* subunit had a complete coding sequence of 657 bp, coding for 218 amino acids with an estimated molecular mass of 24.2 kDa. For *scn4b*, the complete coding sequence was 702 bp, coding for 233 amino acids with an estimated molecular mass of 25.6 kDa.

The deduced amino acid sequences of the Scna and the Scnb isoforms from *E*. *electricus* were compared with those from other animals available in GenBank (S2 Table). Based on the percentage similarities with sequences from other species, the two isoforms of Scn α-subunits from *E*. *electricus* were identified as Scn4aa and Scn4ab (Table 1) while the three β-subunits were identified as Scn1b, Scn2b and Scn4b (Table 2). The *scn4aa*/Scn4aa obtained in this study was verified to be the same as that of the *E*. *electricus* sodium channel (accession no. M22252.1) reported by Noda et al. [20]. A partial Scn4ab sequence (ADQ00363.1) previously reported by Arnegard et al. [42] matches amino acids 576 to 1481 of the complete Scn4ab coding sequence (S1 Fig; 99.4% similarity) obtained in this study.

**Table 1:** Percentage similarity between the amino acid sequence of voltage-gated Na^+^ channel (Scn) type IV α-subunit isoforms (Scn4aa and Scn4ab) of *Electrophorus electricus* as compared with Scn4aa and Scn4ab of other teleosts obtained from GenBank. Sequences are arranged in descending order of similarity. Similarity indices were obtained using the BioEdit software.

Species	*E*. *electricus*	
	Scn4aa	Scn4ab
*E*. *electricus* Scn4aa	ID	59.6%
*E*. *electricus* Scn4ab	59.6%	ID
*Sternopygus macrurus* Scn4aa	**70.9%**	60.9%
*Danio rerio* Scn4aa	**66.0%**	65.8%
*Tetraodon nigroviridis* Scn4aa	**61.0%**	63.0%
*Takifugu rubripes* Scn4aa	**61.1%**	62.4%
*Sternopygus macrurus* Scn4ab	58.0%	**79.5%**
*Danio rerio* Scn4ab	58.3%	**71.4%**
*Tetraodon nigroviridis* Scn4ab	57.6%	**67.6%**
*Takifugu rubripes* Scn4ab	57.2%	**67.3%**

**Table 2:** Percentage similarity between the amino acid sequence of voltage-gated Na^+^ channel (Scn) β-subunit isoforms (Scn1b, Scn2b and Scn4b) of *Electrophorus electricus* in comparison with Scnb/SCNB of other animal species obtained from GenBank. Sequences are arranged in descending order of similarity. Similarity indices were obtained using the BioEdit software.

Species	*E*. *electricus*		
	Scn1b	Scn2b	Scn4b
*E*. *electricus* Scn1b	ID	14.7%	13.2%
*E*. *electricus* Scn2b	14.7%	ID	20.5%
*E*. *electricus* Scn4b	13.2%	20.5%	ID
*Sternopygus macrurus* Scn1b	**90.4%**	14.7%	13.2%
*Danio rerio* Scn1b	**68.7%**	12.1%	14.8%
*Esox lucius* Scn1b	**65.1%**	12.6%	13.6%
*Larimichthys crocea* Scn1b	**64.1%**	15.1%	13.2%
*Oreochromis niloticus* Scn1b isoform X2	**62.5%**	13.8%	12.8%
*Oreochromis niloticus* Scn1b isoform X1	**60.9%**	12.9%	12.8%
*Haplochromis burtoni* Scn1b	**60.5%**	13.8%	12.8%
*Danio rerio* Scn1bb	**58.5%**	10.0%	13.6%
*Mus musculus* Scn1b	**43.4%**	16.7%	14.0%
*Homo sapiens* Scn1b	**42.1%**	16.3%	14.0%
*Astyanax mexicanus* Scn2b	14.8%	**80.7%**	20.5%
*Danio rerio* Scn2b	14.1%	**68.1%**	19.5%
*Haplochromis burtoni* Sc2b	14.3%	**67.2%**	21.5%
*Oreochromis niloticus* Scn2b	14.3%	**66.8%**	21.5%
*Larimichthys crocea* Scn2b	13.7%	**65.4%**	19.4%
*Esox lucius* Scn2b	12.9%	**61.4%**	19.8%
*Homo sapiens* Scn2b	11.9%	**46.3%**	20.2%
*Mus musculus* Scn2b	11.3%	**45.7%**	20.5%
*Danio rerio* Scn4ba	11.6%	20.1%	**79.8%**
*Esox lucius* Scn4b	14.4%	21.8%	**75.1%**
*Haplochromis burtoni* Scn4b	12.8%	23.1%	**73.8%**
*Oreochromis niloticus* Scn4b	12.8%	23.1%	**73.3%**
*Danio rerio* Scn4bb	12.8%	17.0%	**58.2%**
*Mus musculus* Scn4b	15.2%	19.4%	**46.4%**
*Homo sapiens* Scn4b	16.1%	20.6%	**43.8%**
*Xenopus (Silurana) tropicalis* Scn4b	12.2%	19.7%	**37.8%**

### Molecular characterization of Scn4ab

The structure of Scn4aa had been well-analyzed as its full sequence was first reported in the 1980s [20]. In this study, we carried out a characterization of the Scn4ab sequence, comparing it with that of Scn4aa (S2 Fig). Similar to Scn4aa, Scn4ab had four homologous domains (I-IV) with six transmembrane regions (S1-S6) each, making up to a total of 24 transmembrane regions. Key residues which might function as voltage-sensors and for ion selectivity were highlighted. Residues constituting four different hydrophobic clusters which might play important roles in voltage-dependent activation and inactivation were identified and tagged accordingly. The motif responsible for fast inactivation was identified as leucine-phenylalanine-methionine (LFM) with the neighbouring threonine. Residues possibly acting as hinges in gating were also identified.

### Molecular characterization of Scn1b, Scn2b and Scn4b

Alignments of the deduced amino acid sequences of Scn1b (S3a Fig), Scn2b (S3b Fig) and Scn4b (S3c Fig) of *E*. *electricus* with corresponding sequences from *Danio rerio*, *Rattus norvegicus* and *Homo sapiens*, revealed conserved characteristic domains and residues of Scnb/SCNB. Each Scnb subunit had an extracellular Ig loop domain, a single transmembrane region, and an intracellular C-terminal region (S3 Fig). The putative signal peptide sequences were located at the N-terminal regions. Conserved residues important for interactions with Scna and for the maintenance of the overall functional structure of the Ig loop were also identified.

### Evolutionary rates in *scn1b*, *scn2b* and *scn4b* of *E*. *electricus* and non-electric fishes

The topology of the Bayesian tree (S4 Fig) was mostly identical to that of the ML tree, with the exception of one conflicting node. Using the branch lengths as an indication of evolutionary rates of the genes within each gene-specific clade, no clear trend can be interpreted for the *scn2b* and *scn4b* genes between *E*. *electricus* and the non-electric fishes. Similarly, there was no clear pattern to the rates of evolution of *scn1b* from *E*. *electricus* and *Sternopygus macrurus* when compared to non-electric fishes.

### mRNA expression levels of *scna* and *scnb* isoforms in the EOs and the SM of adult and juvenile *E*. *electricus*

**Fig 1:**
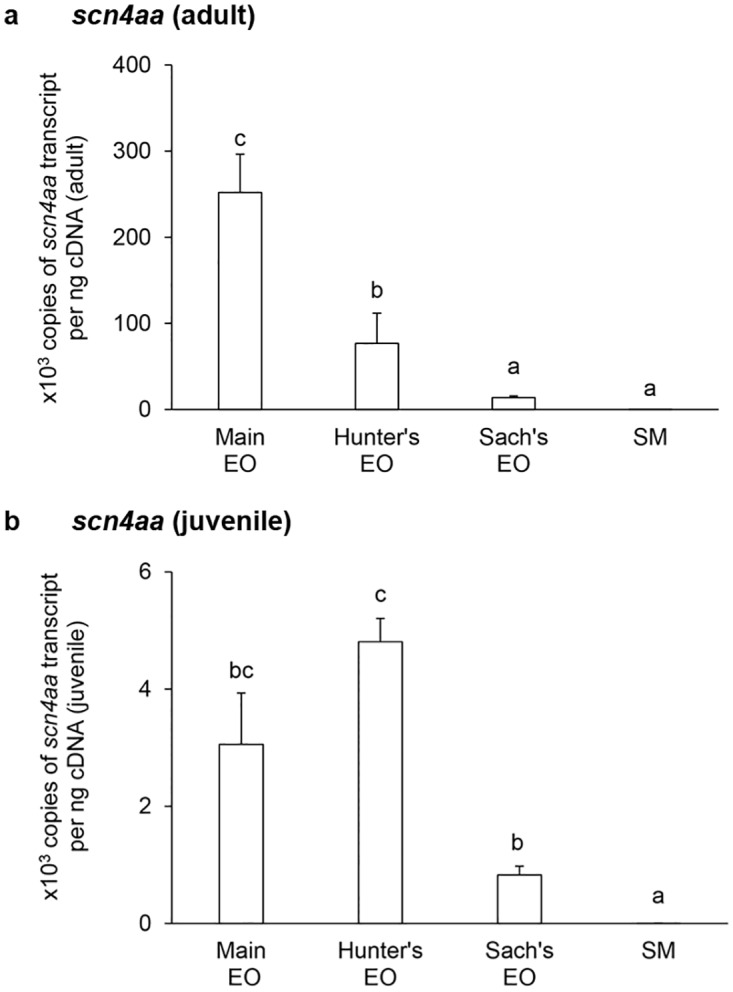
mRNA expression levels of the *voltage-gated Na*^*+*^ *channel type IV α-subunit a isoform* (*scn4aa*) in the electric organs (EOs) and the skeletal muscle (SM) of *Electrophorus electricus*. Absolute quantification (×10^3^ copies of transcript per ng of cDNA) of *scn4aa* transcripts in the main EO, the Hunter’s EO, the Sach’s EO and the SM of (a) adult or (b) juvenile *E*. *electricus* kept in fresh water. Results represent means ± S.E.M. (*N* = 4). Means not sharing the same letter are significantly different (*P*<0.05).

**Fig 2:**
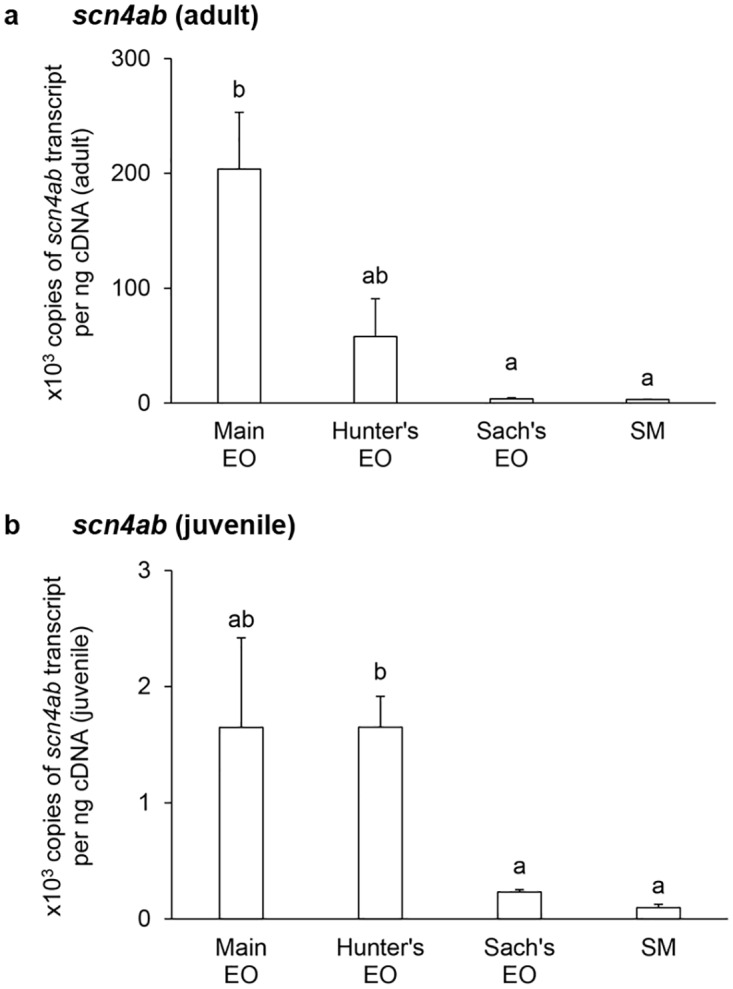
mRNA expression levels of the *voltage-gated Na*^*+*^ *channel type IV α-subunit b isoform* (*scn4ab*) in the electric organs (EOs) and the skeletal muscle (SM) *Electrophorus electricus*. Absolute quantification (× 10^3^ copies of transcript per ng of cDNA) of *scn4ab* transcripts in the main EO, the Hunter’s EO, the Sach’s EO and the SM of (a) adult or (b) juvenile *E*. *electricus* kept in fresh water. Results represent means ± S.E.M. (*N* = 4). Means not sharing the same letter are significantly different (*P*<0.05).

**Fig 3:**
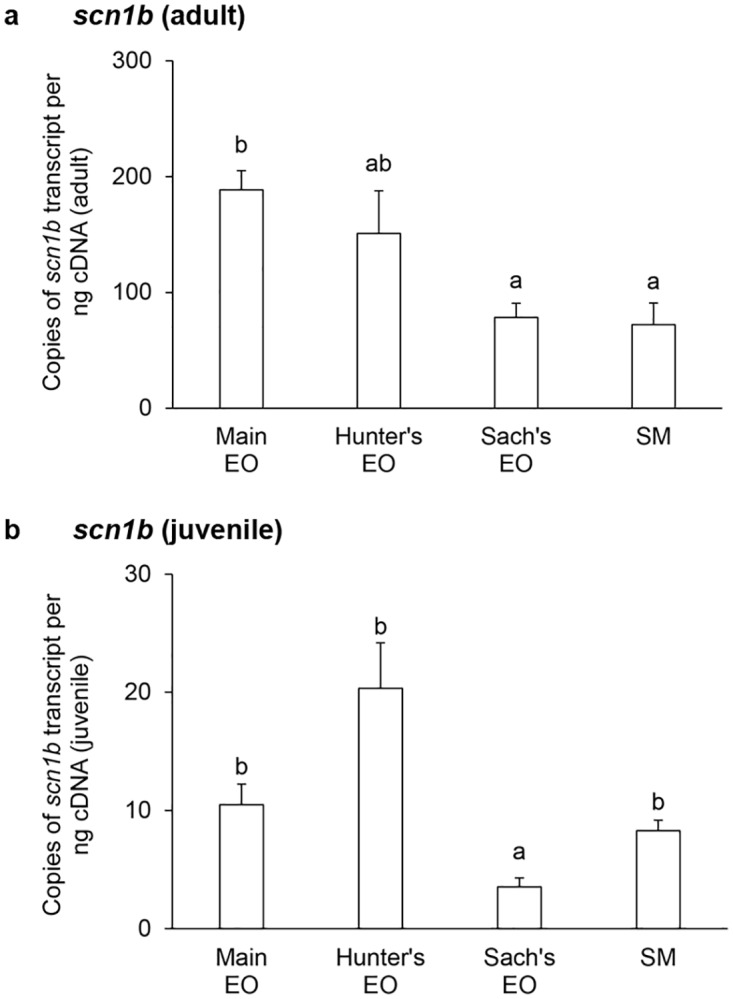
mRNA expression levels of the *voltage-gated Na*^*+*^ *channel β-subunit 1 isoform* (*scn1b*) in the electric organs (EOs) and the skeletal muscle (SM) *Electrophorus electricus*. Absolute quantification (copies of transcript per ng of cDNA) of *scn1b* transcripts in the main EO, the Hunter’s EO, the Sach’s EO and the SM of (a) adult or (b) juvenile *E*. *electricus* kept in fresh water. Results represent means ± S.E.M. (*N* = 4). Means not sharing the same letter are significantly different (*P*<0.05).

**Fig 4:**
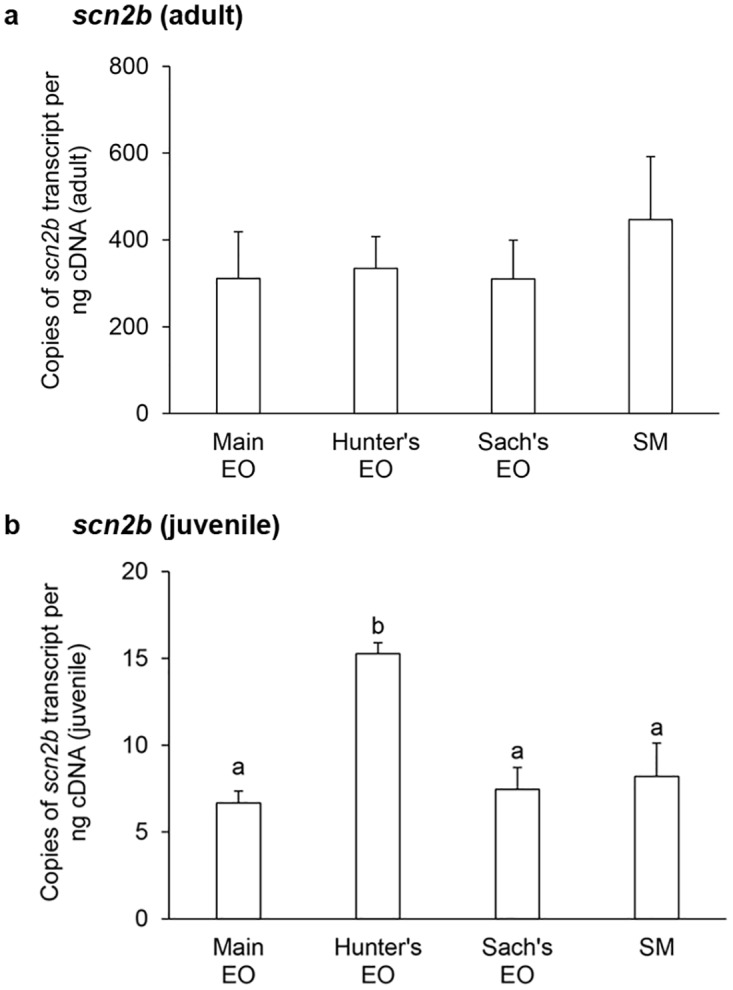
mRNA expression levels of *voltage-gated Na*^*+*^ *channel β-subunit 2 isoform* (*scn2b*) in the electric organs (EOs) and the skeletal muscle (SM) *Electrophorus electricus*. Absolute quantification (copies of transcript per ng of cDNA) of *scn2b* transcripts in the main EO, the Hunter’s EO, the Sach’s EO and the SM of (a) adult or (b) juvenile *E*. *electricus* kept in fresh water. Results represent means ± S.E.M. (*N* = 4). Means not sharing the same letter are significantly different (*P*<0.05).

**Fig 5:**
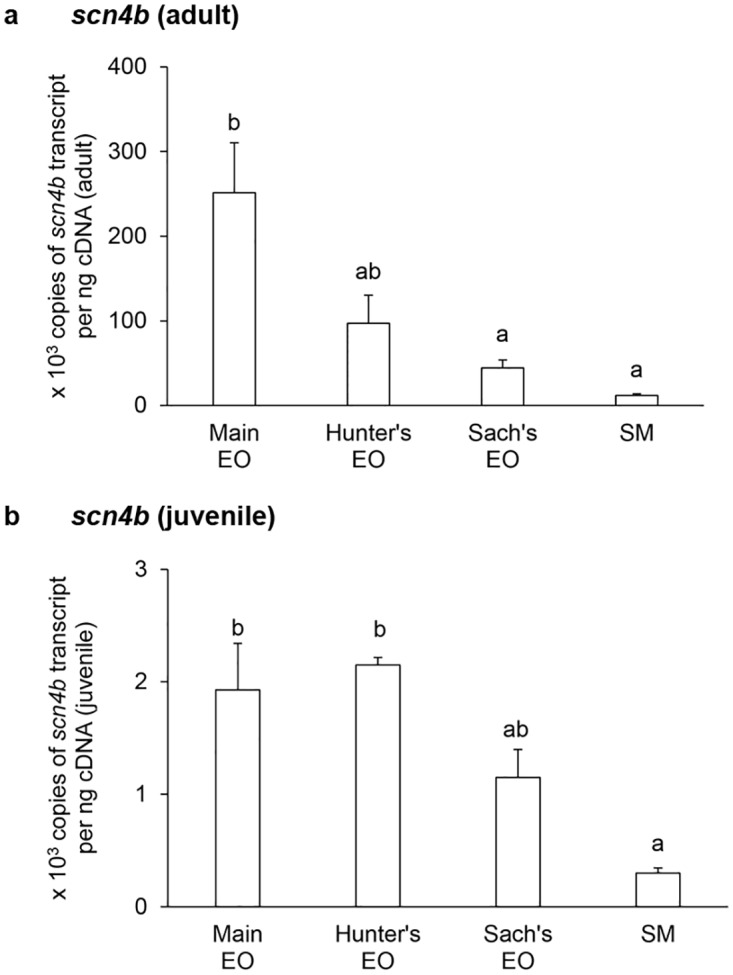
mRNA expression levels of *voltage-gated Na*^*+*^ *channel β-subunit 4 isoform* (*scn4b*) in the electric organs (EOs) and the skeletal muscle (SM) *Electrophorus electricus*. Absolute quantification (× 10^3^ copies of transcript per ng of cDNA) of mRNA expression of *scn4b* in the main EO, the Hunter’s EO, the Sach’s EO and the SM of the (a) adult or (b) juvenile *E*. *electricus* kept in fresh water. Results represent means ± S.E.M. (*N* = 4). Means not sharing the same letter are significantly different (*P*<0.05).

In general, the mRNA expression levels of *scna* (Figs 1 and 2) and *scnb* (Figs 3–5) in the three EOs and the SM of adult *E*. *electricus* (Figs 1a–5a) were much higher (10- to 40-fold) than those of juvenile fishes (Figs 1b–5b).

For adult fish, the transcript level of *scn4aa* was the highest in the main EO and the lowest in the Sach’s EO (Fig 1a). The *scn4aa* transcript levels in the Sach’s EO and the SM were similar and they were significantly lower than those of the main EO and the Hunter’s EO (Fig 1a). For juvenile fish, the SM had a significantly lower *scn4aa* transcript level than the three EOs (Fig 1b). Among the EOs, the Hunter’s EO and the main EO appeared to have the highest expression level of *scn4aa* as they were comparable to each other (Fig 1b). The mRNA expression levels of *scn4ab* (Fig 2a) in the EOs and the SM of adult *E*. *electricus*, and the pattern of expression level among them, were similar to those of *scn4aa* (Fig 1a) in adult *E*. *electricus*. In adult fish, the *scn4ab* transcript levels in the Sach’s EO and the SM were significantly lower than that in the main EO (Fig 2a). In juvenile fish, the *scn4ab* expression in the Hunter’s EO was comparable to that of the main EO, but significantly higher than those of the Sach’s EO and the SM (Fig 2b).

The transcript level of *scn1b* mRNA in the main EO was comparable to that of the Hunter’s EO but significantly higher than those in the Sach’s EO and the SM in adult *E*. *electricus* (Fig 3a). By contrast, the transcript levels of *scn1b* mRNA in the main EO, the Hunter’s EO and the SM were comparable, and the Sach’s EO had the lowest *scn1b* transcript level in juvenile fish (Fig 3b). There were no significant differences in mRNA expression levels of *scn2b* among the three EOs and the SM in adult fish (Fig 4a), but in juveniles, the expression of *scn2b* in the Hunter’s EO was significantly higher than those in other EOs and the SM (Fig 4b). Among the three *scnb* examined, *scn4b* had the highest levels of mRNA expression. In adult fish, the main EO had a significantly higher level of *scn4b* expression than the Sach’s EO and SM (Fig 5a). The transcript levels of *scn4b* in the main and Hunter’s EOs of juvenile fish were comparable, and they were significantly higher than that in the SM (Fig 5b).

### Protein abundances of Scn4ab and Scn4b in the EOs and the SM of adult and juvenile *E*. *electricus*

**Fig 6:**
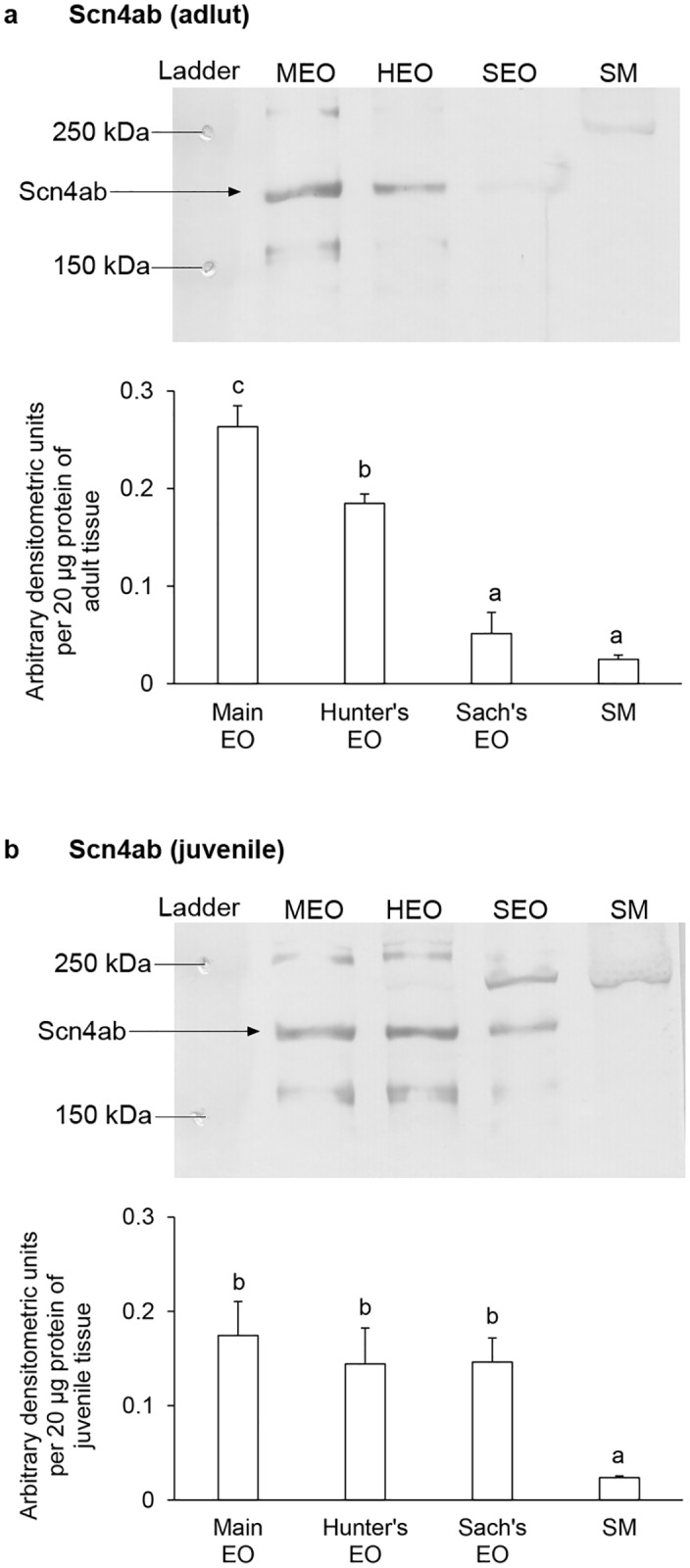
Protein abundances of voltage-gated Na^+^ channel type IV α-subunit b isoform (Scn4ab) in the electric organs (EOs) and the skeletal muscle (SM) of *Electrophorus electricus*. Representative immunoblots and protein abundances of Scn4ab in the main EO, the Hunter’s EO, the Sach’s EO and the SM of (a) adult or (b) juvenile *E*. *electricus*. Protein abundance is expressed as arbitrary densitometric units per 20 μg protein. Results represent mean ± S.E.M (*N* = 3). Means not sharing the same letter are significantly different (*P* < 0.05).

**Fig 7:**
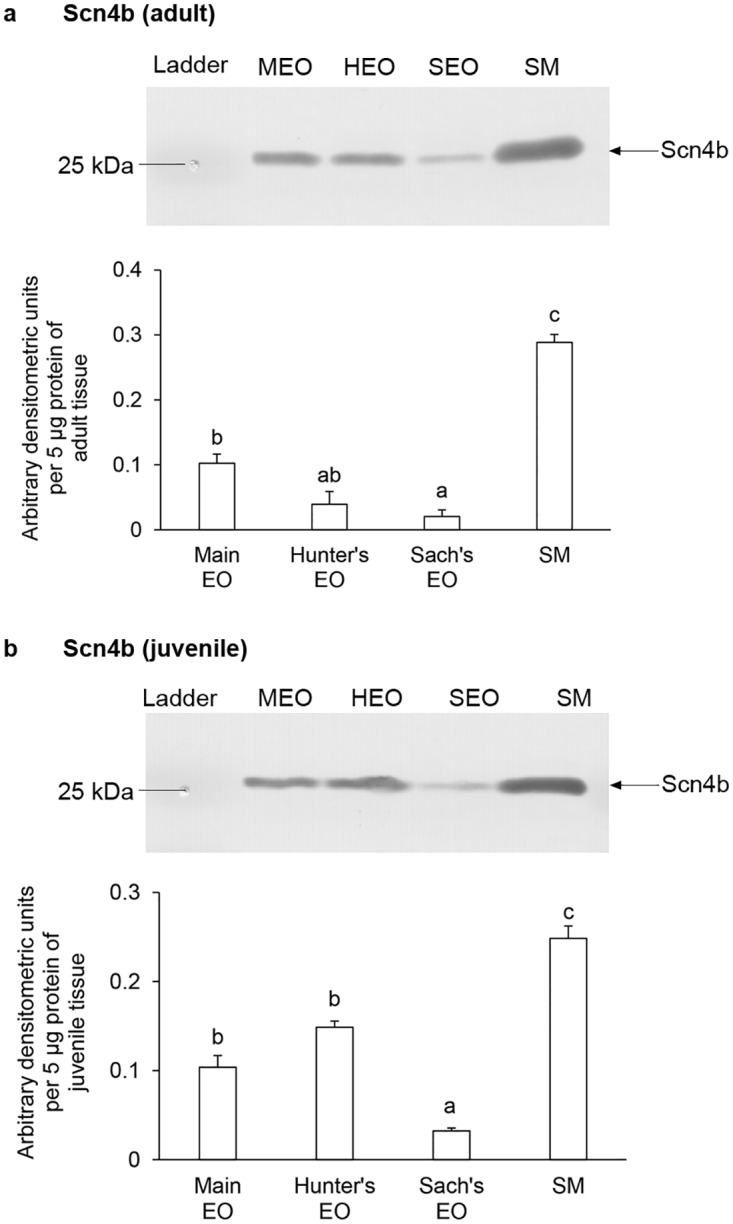
Protein abundances of voltage-gated Na^+^ channel β-subunit 4 isoform (Scn4b) in the electric organs (EOs) and the skeletal muscle (SM) of *Electrophorus electricus*. Representative immunoblots and protein abundances of Scn4b in the main EO, the Hunter’s EO, the Sach’s EO and the SM of (a) adult or (b) juvenile *E*. *electricus*. Protein abundance is expressed as arbitrary densitometric units per 20 μg protein. Results represent mean ± S.E.M (*N* = 3). Means not sharing the same letter are significantly different (*P* < 0.05).

Western blotting revealed a band of Scn4ab at ~200 kDa (Fig 6) and a band of Scn4b at ~25kDa (Fig 7), which were close to their deduced molecular mass. The identities of the bands were validated through blocking peptide competition assays. The protein abundance of Scn4ab in the main EO of adult *E*. *electricus* was the highest, followed by the Hunter’s EO, the Sach’s EO and the SM (Fig 6a). The Scn4ab protein abundance of the Sach’s EO was comparable to that of the SM. For juvenile *E*. *electricus*, all three EOs had significantly higher Scn4ab abundance than the SM (Fig 6b). In both adult and juvenile *E*. *electricus*, Scn4b was highly expressed in the SM compared to the EOs (Fig 7a and 7b), while the main EOs had significantly higher Scn4b expression than the Sach’s EOs (Fig 7a and 7b).

## Discussion

### Molecular characterization of Scn4ab of *E*. *electricus*

The predicted 24 transmembrane regions of Scn4ab were mapped according to the primary structure of Scn4aa of *E*. *electricus* [20]. Most of the sequences in the transmembrane regions are well-conserved in both isoforms, showing the importance of the basic structure in function. The S4 regions of all four domains show the distinct voltage-sensing elements in which there are four or more conserved positively charged arginine or lysine residues, separated by two hydrophobic residues (arbitrarily labelled R1-R4) [43,44].

During activation, the extracellular negative-charge cluster (ENC) and the intracellular negative-charge cluster (INC) are involved in the stabilization and movement of the S4 segments [45,46]. The hydrophobic constriction sites (HCS), which are located in the core of the voltage-sensing domain (VSD), line the narrowest part of the gating charge movement pathway and also form a seal to avoid ion leakage as the S4 segments rotate during activation as proposed in the *helical screw* model [47]. The gating charges interact with ENC when in the activation conformation and the INC when in the inactivation conformation. The hydrophobic charge region (HCR) also interacts via hydrogen bonding with the S4 segments (See [48,49] for comprehensive reviews on Scn channel structure and function). The above-mentioned regions were well-conserved in Scn4aa and Scn4ab of *E*. *electricus*.

The region responsible for fast inactivation is the loop between domain III and IV containing the motif isoleucine-phenylalanine-methionine (IFM) and neighbouring threonine residue. This motif forms the inactivation gate along with conserved glycine residues serving as molecular hinges [50–52]. In both Scn4aa and Scn4ab of *E*. *electricus*, the isoleucine residue is replaced by a leucine residue, forming an LFM motif. The property of leucine is similar to that of isoleucine, except that it is not Cβ branched [53]. This substitution may aid in reducing the bulkiness of the inactivation gate thus affecting inactivation rate in *E*. *electricus*. However, site directed mutagenesis studies will be required to find out the actual effect of this substitution in the future.

SCN/Scn is a target of post-translational regulation, especially phosphorylation and glycosylation. Phosphorylation on the I-II linker has been shown to play an important role in current reduction [54]. The I-II linker region on Scn4aa contains a 51 amino acid insert not found on Scn4ab. An analysis by the NetPhos prediction software indicated a cluster of potential phosphorylation sites in this region which may affect its regulation. The difference in phosphorylation sites on the two isoforms may be important to the regulation of the magnitude of the ionic current, and may indicate different roles of these two isoforms. Glycosylation is also an important post translational modification for all sodium channel subunits [55] and may be important in biosynthesis and degradation of sodium channels [56]. In fact, it has been reported previously that the Scn4aa [Accession: M22252.1] from EOs of *E*. *electricus* is very heavily glycosylated, with carbohydrate accounting for 40% of its mass [21]. N-linked glycosylation adding highly-charged carbohydrates such as sialic acid, can alter the surface charge of the sodium channel protein, thereby affecting the voltage dependence of gating in the channel [55]. Out of several identified N-glycosylation sites in Scn4aa of the electric eel, two of the sites on Scn4aa (N278 and N591) were substituted by a serine and threonine on Scn4ab (S286 and T549, respectively). This indicates that post-translational regulation differs for these two isoforms, which may result in slight differences in properties even though key residues and motifs are highly conserved between the isoforms.

### Molecular characterization of Scnb isoforms of *E*. *electricus*

SCNB/Scnb are single-pass type I membrane proteins, consisting of one transmembrane region, an extracellular immunoglobulin (Ig) loop at the N-terminal, and an intracellular C-terminal region [57]. The Ig loop is characteristic of the cell adhesion molecules (CAMs), and SCNB/Scnb are indeed found have CAM-like functions [58,59]. There are several cysteine residues typically conserved within the Ig loop, forming disulphide bridges which hold the β-sheets forming the structure of the loop. In human SCN4B, a disulphide bridge buried in a hydrophobic pocket formed between C53 and C131 is important for maintaining the local conformation for interaction with SCNA [60]. These two cysteine residues are conserved for all SCNB/Scnb of various animal species compared in this study. The C55 site in the human SCN2B mediates the disulphide linkage to SCNA [61]. This cysteine is conserved in SCN4B (C58) as well, probably serving as a common docking site for different SCNA isoforms [60]. Correspondingly, the analogous cysteine sites were conserved in the *E*. *electricus* Scn2b and Scn4b, indicating that these isoforms do contain the universal docking site for Scna, and have the capability to interact with it. Several charged residues in rat SCN1B (23E, 25D, 27E) were found to be essential for interacting with and modulating the activity of SCNA [62]. In *E*. *electricus* Scn1b, charged residues were also found at the corresponding sites (27E, 29D, 30D), indicating the putative site for association with Scn4aa and Scn4ab.

Chopra et al. [63] and Liu et al. [64], working on zebrafish and electric fish, respectively, were among the first to report the existence of Scna-Scnb complexes in non-mammalian vertebrates, when previous studies suggested that these complexes can only be found in mammals. Hence, Chopra et al. [63] called for further investigations into other non-mammalian vertebrates to consolidate and verify information on this issue. Since this study discovered three isoforms of Scnb, whereas prior studies only managed to identify two Scna isoforms from *E*. *electricus* [20,42,65,66], the involvement of and interaction between the various Scna and Scnb isoforms in bioelectrogenesis in *E*. *electricus* needs to be re-evaluated.

### Differential expression of *scn4aa* and *scn4ab*/Scn4ab in the EOs and the SM of adult *E*. *electricus*

There is a large diversity of SCNA, some of which are predominantly expressed in certain organ/tissue [67]. For example, Scn1a, Scn2a, Scn3a and Scn8a are found mostly in the central nervous system, Scn9a is found in the peripheral nervous system while Scn10a and Scn11a are found in posterior root ganglion neurons. Scn5a is primarily found in the cardiac muscles, while Scn4a is expressed mainly in SM [67].

The expression of *scn4aa* and *scn4ab* in the EOs and the SM of *E*. *electricus* supports the hypothesized myogenic origins of the EOs. Studies on the evolution of the EOs in electric fish showed that *scn4aa* was lost from the SM but neo-functionalized in EOs, while *scn4ab* remained expressive in the SM [42,68,69]. Accordingly, our results show that the number of copies of *scn4aa* transcripts in the SM (11 copies per ng cDNA) was close to undetectable, while it was significantly higher in the EOs. In addition, the mRNA expression of *scn4ab* was much higher than that of *scn4aa* in the SM of both adult and juvenile *E*. *electricus*. This thus reinforces the postulate that *scn4aa* expression had been lost from the SM of electric fishes and that Scn4aa is an EO-predominant form. Several studies have previously raised antibodies against Scn4aa of *E*. *electricus* and conducted Western blot and/or immunohistochemical work on the EO and the SM [16,29,30]. These studies consistently found intense immunoreactivity on the innervated side of the electrocyte [16,29,30]. Furthermore, Fritz and Brockes [16] reported faint or undetectable staining of Scn4aa in the SM as compared to the staining on the innervated face of electrocytes from the Sach’s EO. This further shows that protein expression of Scn4aa is predominant in the EO, corroborating the mRNA expression results in this study. In another study, immunofluorescence of Scn4aa in the SM was localized to the T-tubules and sarcolemma [29]. However, it must be noted that the antibodies designed in some of these studies might not be able to differentiate between Scn4aa and Scn4ab, as only one isoform in *E*. *electricus* was known at that time. This could have contributed to the varying immunoreactivity results in the SM for different studies in the 1980s. As the mRNA expression level of *scn4aa* is the highest in the main EO of adult *E*. *electricus*, it probably plays an important role in the production of high voltage EODs. A transcriptome analysis done on *E*. *electricus* [70] found *scn4aa* to be highly expressed in the EOs, giving further support to our data.

Although *scn4ab* is also known to be expressed in the EOs of electric fishes, unlike *scn4aa*, there was apparently a strong negative selection due to constraints imposed on it by its continued expression in the SM [42]. Scn4ab has thus been associated largely with muscle functions, while Scn4aa is the focus of studies on bioelectrogenesis. To our knowledge, there have been no studies relating or examining the role of *scn4ab*/Scn4ab in bioelectrogenesis in *E*. *electricus*. Our results demonstrate for the first time the high mRNA expression level of *scn4ab* in the EOs of *E*. *electricus*, with expression levels comparable to those of *scn4aa* in the main EO and the Hunter’s EO of the adult fish. The mRNA and protein expression levels of *scn4ab*/Scn4ab in the EOs were generally higher than those in the SM. This, coupled with the fact that key domains affecting the properties of the ion channel were mostly conserved between Scn4aa and Scn4ab (see discussion above), suggests that Scn4ab may have a functional role in electrogenesis as well. So far, there is no study on the subcellular localization of Scn4ab in EOs of electric fishes, but both Scn4aa and Scn4ab are known to be important for the development of neural and muscle tissues, and have been found to be expressed in non-overlapping tissues throughout the development of zebrafish embryo [71]. Therefore, it is logical to deduce that *scn4aa* and *scn4ab* may be partitioned to different cells or subcellular localizations in the EOs of *E*. *electricus*, and they may have different sub-functions in electrogenesis.

With three EOs, *E*. *electricus* is capable of producing both strong and weak EODs. The Sach’s EO, which exclusively produces low voltage EODs, would be considered the most similar to EOs of other weakly-electric fishes. Expression patterns of *scn4ab* in other weakly electric fishes mostly showed weaker expression in the EOs than SM [42, 68]. The exceptions are *Rhamphichthys marmoratus*, which shows stronger expression of *scn4ab* in the EO than SM [42], and *Brachyhypopomus pinnicaudatus* which displays similar expression between both tissues [68]. In comparison, our results indicate low and comparable levels of *scn4ab* transcripts in the Sach’s EO and SM of *E*. *electricus*. The low-voltage EOD produced by the Sach’s EO of *E*. *electricus* (1–10 V) is at least 10 times stronger than a typical gymnotiform EOD [8] or EODs (<1 V) of other weakly electric fishes. If Scn4ab indeed has a functional role in electrogenesis and is related to the strength of the EOD produced, the Sach’s EO of *E*. *electricus* should have a comparatively higher expression of *scn4ab*/Scn4ab than the EOs of weakly electric fishes. Unfortunately, the above-mentioned studies [42, 68] performed only semi-quantitative estimates of *scn4ab* expression without statistical analyses in weakly-electric fishes, which do not allow a direct comparison with results obtained for *E*. *electricus* in this study. More quantitative data obtained through qPCR on mRNA expression levels of *scn4ab* in EO versus SM are needed for a better cross-species comparison in order to evaluate the possible role of Scn4ab in electrogenesis.

### Differential expression of *scn1b*, *scn2b* and *scn4b*/Scn4b in the EOs and the SM of adult *E*. *electricus*

Although SCNB/Scnb isoforms do not form the ion channel, they hold critical roles in channel modulation by affecting the voltage-dependence, gating and kinetics of SCNA/Scna [57]. There are four known genes (*SCN1B* to *SCN4B*) encoding for five SCNB (SCN1B, SCN1BB, SCN2B, SCN3B and SCN4B) in mammalian tissues [57]. In this study, three out of the four *scnb* have been identified for the first time in the EOs and the SM of *E*. *electricus*.

SCN1B is known to alter the functional properties of SCNA [72]. The absence of SCN1B reduces the speed of conduction of action potentials in the optic nerves of mice [73], and increases the peak voltage and current of action potentials in CA3 neurons [74]. Both SCN1B and SCN2B from rat brain neurons have been shown to increase current and inactivation when co-expressed with SCNA in *Xenopus* oocytes [58]. In adult *E*. *electricus*, the transcript levels of *scn2b* in the EOs and the SM were comparable; thus, unlike Scn4aa and Scn4ab, Scn2b might not have a specialized function in electrogenesis. SCN2B is expressed mainly in the heart and the central and peripheral nervous systems [57], and thus may partake in basic nervous functions in the tissues of *E*. *electricus*. On the other hand, *scn1b* is expressed significantly higher in the main EO than the Sach’s EO and SM, suggesting a possible involvement in the regulation of Scn4aa and/or Scn4ab function during the production of high voltage EOD. In *S*. *macrurus*, a higher expression of *scn1b*/Scn1b in the EO had been found to correlate with high EOD frequency-producing fish, giving further support for Scnb being involved in the fine-tuning of cellular excitability [64].

The level of mRNA expression of *scn4b* was much higher than those of *scn1b* and *scn2b* in the EOs and the SM of *E*. *electricus*. SCN4B is mainly expressed in excitable tissues of mammalian systems, and in the SM, SCN4B can covalently bond with SCN4A to increase the membrane sensitivity to action potentials [59]. SCN4B can also override the functions of SCN1B and SCN3B in shifting the voltage-dependence of activation to a more negative potential [59]. This could also be the case in *E*. *electricus*, and the high expression of *scn4b* above *scn1b* and *scn2b* emphasizes its importance in tissue functions. The mRNA expression of *scn4b* in *E*. *electricus* was the highest in the main EO, but the protein abundance was the highest in the SM instead. Among EOs, the trend of Scn4b protein expression corroborates that of mRNA expression, with the main EO being significantly higher than the Sach’s EO. The lack of correlation between mRNA and protein expression for Scn4b in the SM could be due to tissue specific transcription and translational controls, or different mechanisms of protein synthesis and degradation employed in the tissue [75]. SCNB/Scnb have additional functions besides modulating electrical excitability [57], such as activities associated to cell adhesion [58,59], homophilic cell interactions [76,77] or neurite development [78,79]. Since SCN4B/Scn4b are involved in a range of functions, the high protein expression of Scn4b in the SM compared to the functionally-distinct EOs in *E*. *electricus* is not unexpected. The relatively higher expression of *scn4b*/Scn4b in the main EO and Hunter’s EO capable of generating high EODs suggests that Scn4b may be the main Scnb modulating high EOD electrogenesis. However, as each Scnb isoform ma have complex associations with different Scna in specific cell types [57,59], it is probable that different combinations of Scn4aa/Scn4ab and various Scnb isoforms in the three EOs may account for the differences in EODs produced in *E*. *electricus*.

### Evolutionary rates in *scn1b*, *scn2b* and *scn4b* of *E*. *electricus* and non-electric fishes

Several studies have reported increased evolutionary rates of *scn4aa* compared to *scn4ab* in electric fishes [38,42,68]. Hence, it would be interesting to elucidate whether the *scnb* isoforms, which modulate the Scna channels, would display similar patterns in their evolutionary rates following the evolution of EOs. Based on a rudimentary *scnb* phylogenetic tree constructed (S4 Fig) using the *E*. *electricus* sequences obtained from this study, there seems to be no clear trend on the rates of evolution of the *scnb* isoforms, as indicated by branch lengths, among *E*. *electricus* and non-electric fishes. Considering the limited availability of *scnb* sequences from electric fishes at present, more detailed phylogenetic analyses should be performed when more information becomes available in the future.

### A comparison of expression of *scn*/Scn isoforms between juvenile and adult *E*. *electricus*

For *E*. *electricus*, the transcript levels of various *scn* isoforms in the EOs and the SM were generally higher in adult than in juvenile. While no study has been performed on the *scn* expression during the development of juvenile to adult *E*. *electricus*, differences in spatial and temporal expression of *scn4a* isoforms is known to occur in zebrafish larvae [71], suggesting that they have specific roles in development. In this study, the main EO of adult *E*. *electricus* had significantly higher *scn4aa* mRNA expression level than the Hunter’s EO, but in juvenile fish, there was no significant difference in expression between the main and Hunter’s EOs. Similarly, the three EOs of juvenile fish had comparable protein abundances of Scn4ab, but there were significant differences in protein abundances of Scn4ab among the three EOs of adult fish. In short, the EOs of the adult fish displayed more defined and significant differences in *scn4aa* and Scn4ab expression as compared with the juvenile, which implies that the three EOs of the juvenile fish could be functionally indistinct, especially between the main EO and the Hunter’s EO.

In addition, the transcript levels of *scn1b* and *scn2b* were very low in juvenile *E*. *electricus*, with the highest expression being found in the Hunter’s EO. The Hunter’s EO of juvenile *E*. *electricus* also expressed relatively high transcript and protein levels of *scn4b*/Scn4b. The sequence of physical development of the three EOs in *E*. *electricus* is the Sach’s EO followed by the main EO and then the Hunter’s EO [6]. As the Hunter’s EO takes the longest time to mature, and as Scnb are involved in development and organogenesis [57], the relatively higher expression of *scn1b* and *scn2b* in the Hunter’s EO of juvenile fish could indicate a role in the development of the organ. The subsequent change in expression patterns in the adult organs may represent a shift in the role of these subunits from organ development to encompass other roles such as Scna modulation or cell interactions.

## Conclusion

We have obtained the full coding sequences of *scn4aa*, *scn4ab*, *scn1b*, *scn2b* and *scn4b* from the EOs and the SM of *E*. *electricus*. Both *scn4aa* and *scn4ab* were highly expressed in the EOs, while in the SM, *scn4aa* was barely detectable compared to *scn4ab*. Although electrogenesis in *E*. *electricus* has been attributed to the *scn4aa* isoform in the electrocytes, the high expression of *scn4ab*/Scn4ab in the EOs indicates a possible role in electrogenesis as well. The major *scnb* isoform expressed in the EOs and SM was *scn4b*. Its mRNA and protein expression in the EOs indicate a possible role in electrogenesis of high voltage EODs, in addition to organ developmental functions. The patterns of *scn*/Scn expression in the EOs of juvenile *E*. *electricus* indicate that they could be functionally indistinct at that stage of development.

## Supporting Information

S1 Fig Alignment of the partial sequence of Scn4ab (ADQ00363.1) to the complete coding sequence (CDS) of Scn4ab of *Electrophorus electricus* obtained from this study. (TIF)

S2 Fig Molecular characterization of voltage-gated Na^+^ channel (Scn) type IV α-subunit isoforms (Scn4aa and Scn4ab) of *Electrophorus electricus*. A multiple amino acid alignment of Scn4aa and Scn4ab of *E*. *electricus*. Identical or strongly similar amino acids are indicated by shaded residues. The four predicted homologous domains (I-IV) and six transmembrane segments in each domain (S1-S6) are underlined. The hydrophobic LFM motif is double underlined. Hash tags indicate possible residues that act like hinges in gating. ENC: extracellular negative-charge cluster; INC: intracellular negative-charge cluster; HCS: hydrophobic constriction sites; HCR: hydrophobic charge region. R1-R4: conserved positively charged arginine or lysine residues in voltage sensing domain.
(TIF)

S3 Fig Molecular characterization of voltage-gated Na^+^ channel (Scn) β-subunit isoforms (Scn1b, Scn2b and Scn4b) of *Electrophorus electricus*. Multiple amino acid alignments of (a) Scn1b, (b) Scn2b and (c) Scn4b of *E*. *electricus* with the corresponding Scn1b/SCN1B, Scn2b/SCN2B and Scn4b/SCN4B sequences from selected vertebrate species (*Danio rerio*, *Rattus norvegicus* and *Homo sapiens*). Identical or strongly similar amino acids are indicated by shaded residues. The predicted signal peptide sequences are indicated by dotted lines. The Ig loop domains are underlined, and the transmembrane regions are double-underlined. Residues involved in interactions with the Scna/SCNA are indicated by asterisks. S denotes cysteine residues used for disulphide linkages, and S* denotes cysteine residues involved in interactions with the Scna/SCNA via disulphide linkages.
(TIF)

S4 Fig Phylogenetic tree based on the nucleotide coding sequences of *scn1b*, *scn2b* and *scn4b*. Tree topology and branch lengths correspond to Bayesian inferences. Numbers at each node represent bootstrap support values (based on 1000 bootstraps) and Bayesian posterior probabilities.
(TIF)

S1 File Alignment used for the *scnb* phylogenetic analysis. (FAS)

S1 Table Primers sequences used for PCR, RACE and sequencing. (DOCX)

S2 Table Amino acid sequences or translated nucleotide sequences of Scn/SCN obtained from Genbank or UniProtKB/TrEMBL and their accession numbers used in classification tables and multiple sequence alignments. (DOCX)

S3 Table Primer sequences used for qPCR. (DOCX)

S4 Table Nucelotide coding sequences of *scn* obtained from Genbank or Ensembl and their accession numbers used for phylogenetic analysis. (DOCX)
