# Supplementary figures and images for: Voltage-Gated Na+ Channel Isoforms and Their mRNA Expression Levels and Protein Abundance in Three Electric Organs and the Skeletal Muscle of the Electric Eel Electrophorus electricus
Source: PLoS One. 2016 Dec 1;11(12):e0167589. doi: 10.1371/journal.pone.0167589 (PMC5132174; doi:10.1371/journal.pone.0167589)

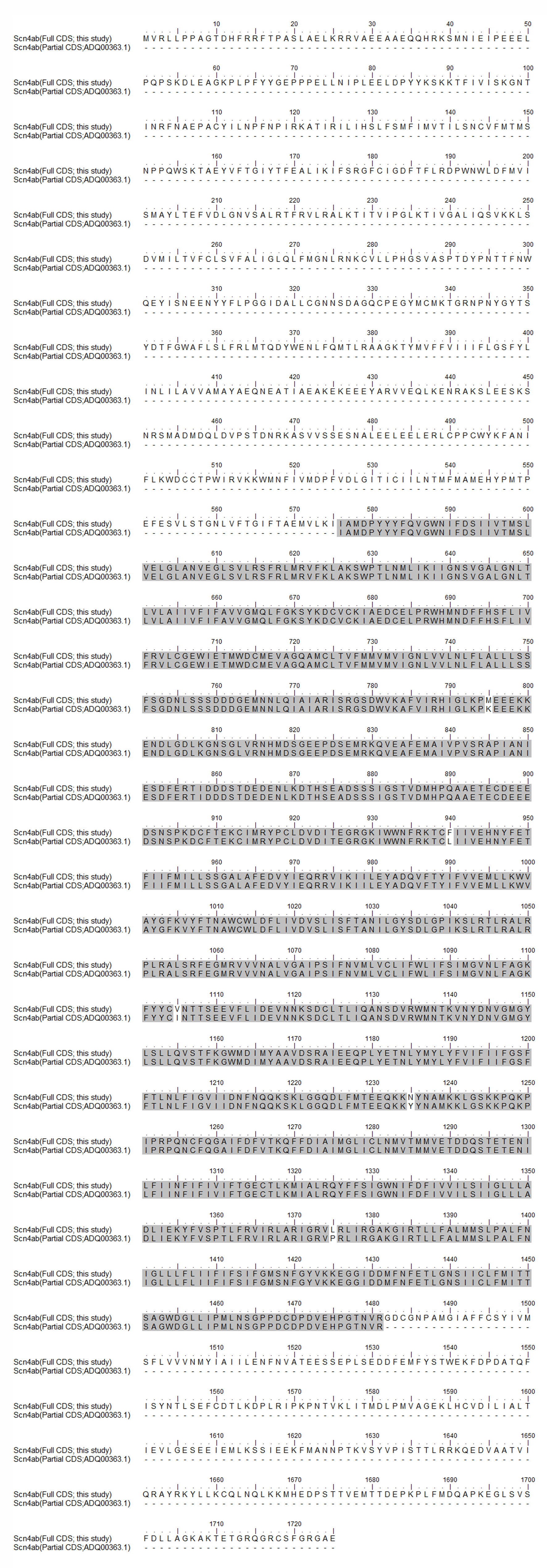

Supplement: S1 Fig — (TIF) [file pone.0167589.s001.tif]

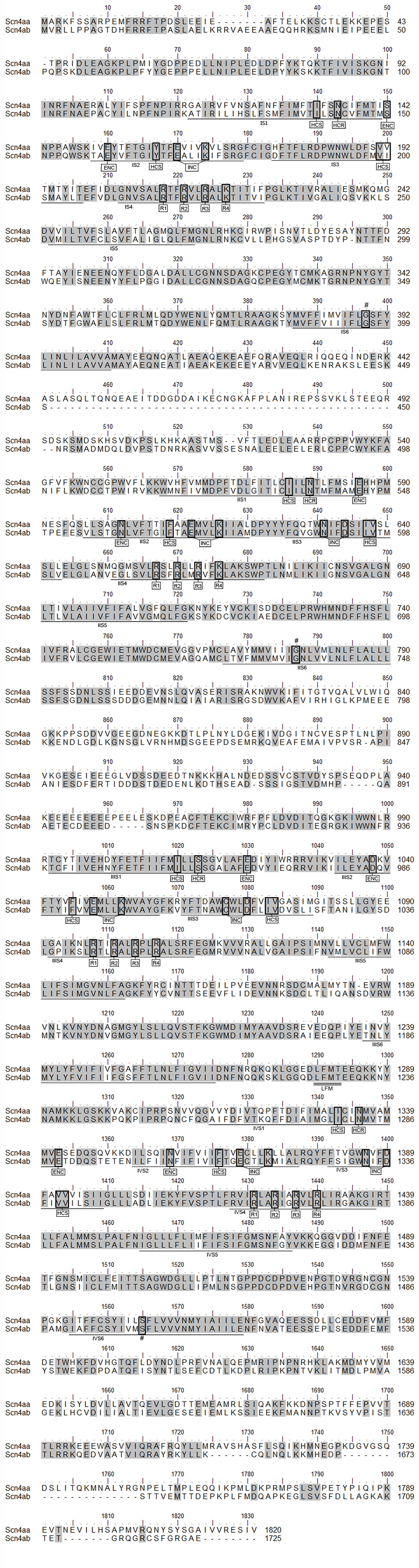

Supplement: S2 Fig — A multiple amino acid alignment of Scn4aa and Scn4ab of E. electricus. Identical or strongly similar amino acids are indicated by shaded residues. The four predicted homologous domains (I-IV) and six transmembrane segments in each domain (S1-S6) are underlined. The hydrophobic LFM motif is double underlined. Hash tags indicate possible residues that act like hinges in gating. ENC: extracellular negative-charge cluster; INC: intracellular negative-charge cluster; HCS: hydrophobic constriction sites; HCR: hydrophobic charge region. R1-R4: conserved positively charged arginine or lysine residues in voltage sensing domain. (TIF) [file pone.0167589.s002.tif]

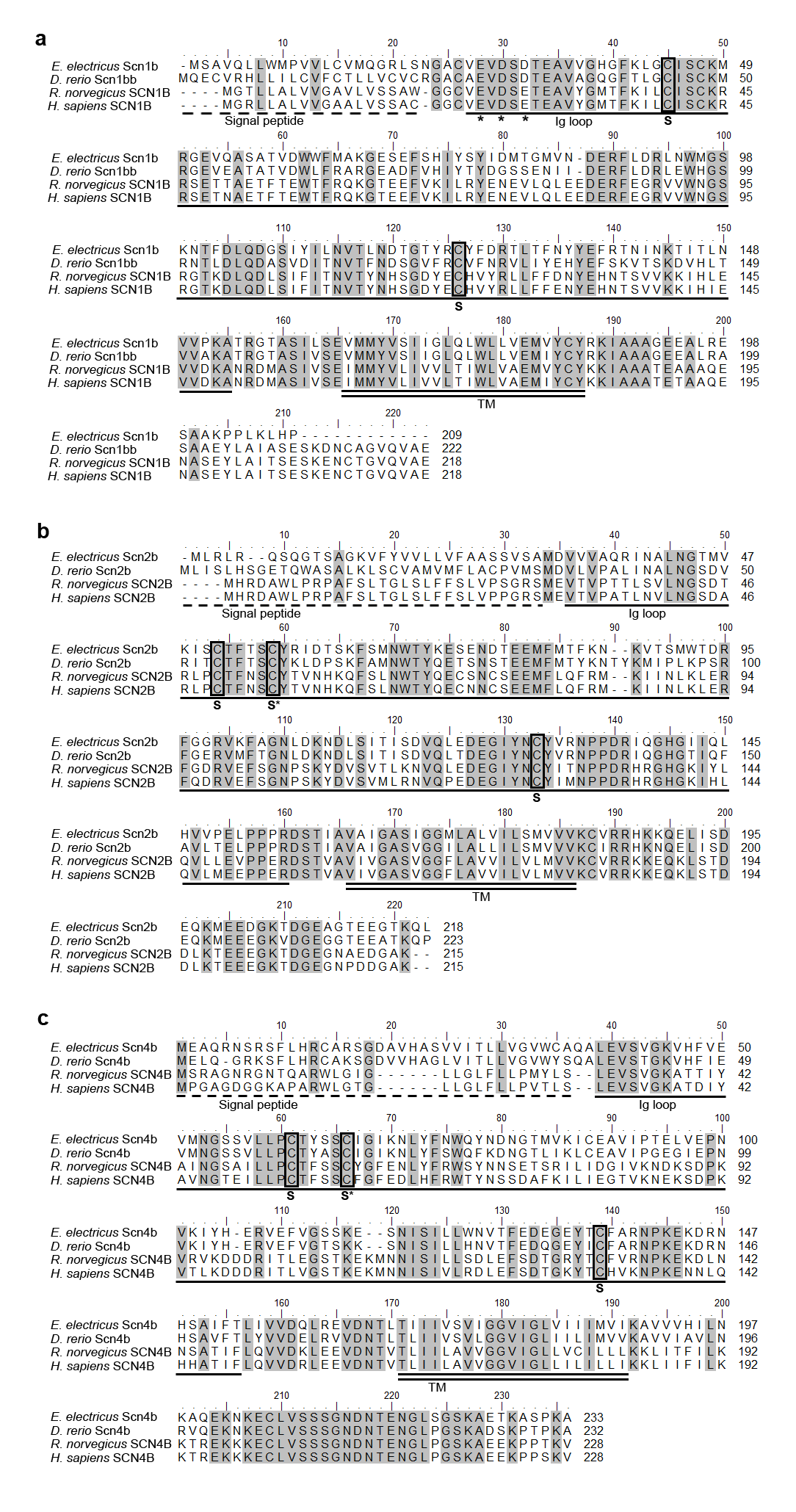

Supplement: S3 Fig — Multiple amino acid alignments of (a) Scn1b, (b) Scn2b and (c) Scn4b of E. electricus with the corresponding Scn1b/SCN1B, Scn2b/SCN2B and Scn4b/SCN4B sequences from selected vertebrate species (Danio rerio, Rattus norvegicus and Homo sapiens). Identical or strongly similar amino acids are indicated by shaded residues. The predicted signal peptide sequences are indicated by dotted lines. The Ig loop domains are underlined, and the transmembrane regions are double-underlined. Residues involved in interactions with the Scna/SCNA are indicated by asterisks. S denotes cysteine residues used for disulphide linkages, and S* denotes cysteine residues involved in interactions with the Scna/SCNA via disulphide linkages. (TIF) [file pone.0167589.s003.tif]

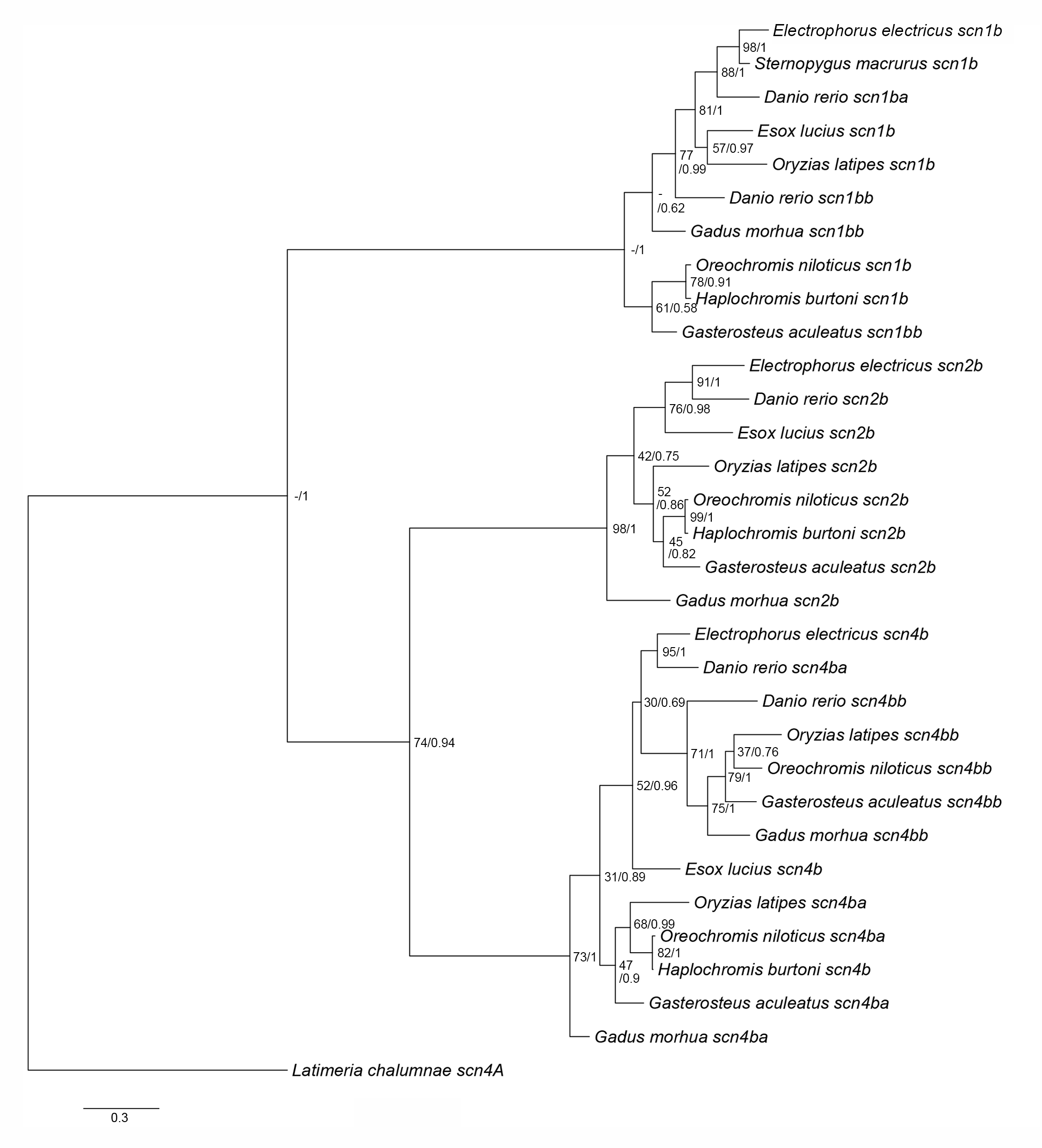

Supplement: S4 Fig — Tree topology and branch lengths correspond to Bayesian inferences. Numbers at each node represent bootstrap support values (based on 1000 bootstraps) and Bayesian posterior probabilities. (TIF) [file pone.0167589.s004.tif]
