# Supplementary material for: Voltage-Gated Na+ Channel Isoforms and Their mRNA Expression Levels and Protein Abundance in Three Electric Organs and the Skeletal Muscle of the Electric Eel Electrophorus electricus
Source: PLoS One. 2016 Dec 1;11(12):e0167589. doi: 10.1371/journal.pone.0167589 (PMC5132174; doi:10.1371/journal.pone.0167589)
Supplement: S1 Table — (DOCX) [file pone.0167589.s006.docx]

**S1 Table.** Primers sequences used for PCR, RACE and sequencing.

| Gene | Primer Name | Primer sequence (5’ to 3’) |
| --- | --- | --- |
| *scn4aa* | Navaa_1284_PCR_F1-1 | AGTCTGCAACATTGATCTTCTG |
|  | Navaa_1284_PCR_R1-1 | GGCTGTAGTTGCTATGGC |
|  | Navaa_1284_PCR_F1-2 | AATGTTTCGCCGCTTCAC |
|  | Navaa_1284_PCR_R1-2 | GAGGAGCAGAATCAGGCAA |
|  | Navaa_1284_PCR_F2-1 | ATCATGGTCATATTCCTGGGT |
|  | Navaa_1284_PCR_R2-1 | CAGCGACAATCTCAGTTCAATT |
|  | Navaa_1284_PCR_F2-2 | CACAAACTATGACAACTTTGCC |
|  | Navaa_1284_PCR_R2-2 | GTGGCGAGTGGATTGAGA |
|  | Navaa_1284_PCR_F3-1 | AACCTGACCATTGTGCTG |
|  | Navaa_1284_PCR_R3-1 | CAGTTTGTTGGGCTATGAAG |
|  | Navaa_1284_PCR_F3-2 | TATCGTCTTCATCTTCGCCT |
|  | Navaa_1284_PCR_R3-2 | AGGTGGTAGTGAGAGCATTG |
|  | Navaa_1284_PCR_F4-1 | GCTCGACTTTGTAATTGTTGG |
|  | Navaa_1284_PCR_R4-1 | GGTCTTCTGCTATCGGACATA |
|  | Navaa_1284_PCR_F4-2 | TTAAGTGGGTTGCATATGGG |
|  | Navaa_1284_PCR_R4-2 | CCCTGTTTCGTGTCATCC |
|  | Navaa_1284_PCR_F5-1 | GTGGCTATGATGGTCGAG |
|  | Navaa_1284_PCR_R5-1 | CCTATGTTAGACAAACCCAGAA |
|  | Navaa_1284_PCR_F5-2 | AACATGGTGGCTATGATGG |
|  | Navaa_1284_PCR_R5-2 | CCTTTCTGAGCCAAATCAAG |
|  | Navaa_1284_PCR_F6-1 | CCAAGTTCAAGAAAGACAACC |
|  | Navaa_1284_PCR_R6-1 | TCGGATATTTGTAGCAGCAC |
|  | Navaa_1284_PCR_F6-2 | CTGGATGTTCTATTGGCTGTC |
|  | Navaa_1284_PCR_R6-2 | CCTTCATTTCATCTAACCACAC |
| *scn4ab* | Nav1.4b_8400_PCR_F1-1 | GAAATTACAGCTACTCCAGGAC |
|  | Nav1.4b_8400_PCR_R1-1 | GGTGGTATTGATGCACTTC |
|  | Nav1.4b_8400_PCR_F2 | ACATCCCTCGTCAAGCAG |
|  | Nav1.4b_8400_PCR_R2 | GTGAATGAACTGGTGGATGG |
|  | Nav1.4b_8400_PCR_R3 | GGATGAACACGAAGGTCAAC |
|  | Nv14b_1991_3RACE_P3 | TTGAGGATGTGTACATTGAGCAACGACGA |
|  | Nv14b_8400_3RACE _P1 | GCCTTATCCAGATTTGAGGGAATGAGGGT |
|  | Nav1.4b_8400_seq_F1 | TGCCTCAGTGTCTTTGCC |
|  | Nav1.4b_8400_seq_F2 | GAAGAGTATGCCAGAGTCGT |
|  | Nav1.4b_8400_seq_F3 | CAGCACAGACAACAGGAAA |
|  | Nav1.4b_8400_seq_F4 | ATTGTGACGATGAGTTTGGTG |
|  | Nav1.4b_8400_seq_F5 | AAAGGCAACAGTGGTCTAGTC |
|  | Nav1.4b_8400_seq_F6 | GACTTGCTTCATCATTGTGG |
|  | Nav1.4b_8400_seq_F7 | AGATTCCAACAGCCCAAAG |
| *scn1b* | Navba_15676_PCR_F1 | CAACTCCTCAGTGAAGATGTC |
|  | Navba_15676_PCR_R1 | CTGTGTTGAGGTATGGACTTT |
|  | Navba_15676_PCR_F2 | TAAACCAAAGAGACAGACTCCT |
|  | Navba_15676_PCR_F3 | ACTGGCGGAATGATTTAAG |
|  | Navba_31765_PCR_F1 | ACTGGCGGAATGATTTAAG |
|  | Navba_31765_PCR_R1 | CTGTGTTGAGGTATGGACTTT |
|  | Navba_31765_3RACE_TD_P1 | GTTGGTGGAGATGGTCTACTGCTACAGG |
| *scn2b* | NvBb_54714_PCR_F2 | GTCAAGATCTCCTGCACCT |
|  | NvBb_54714_PCR_R2 | GTGAAATGCGTGCGTAGG |
|  | NvBb_33291_PCR_F1 | ACCTGTCCATCACCATCTC |
|  | NvBb_33291_PCR_R1 | TCCTTTGGTTACACTGAGCA |
|  | NvBb_33291_PCR_F2 | AATGACCTGTCCATCACCA |
|  | NvBb_33291_PCR_R2 | CTCTTTCCTTTGGTTACACTGA |
|  | NvBb_33291_PCR_R3 | TGGTGTGGGAAACATCCTAAA |
|  | NvBb_54714_5RACE_TD_P1 | TCCTGCACCTTCACTTCCTGCTATCGA |
|  | NvBb_54714_5RACE_TD_P2 | CCATCACCATCTCAGACGTGCAGC |
| *scn4b* | Navba_41335_PCR_F1 | TTTGTTGAGGTGATGAACGG |
|  | Navba_41335_PCR_R1 | CAGAAACCAAAGCATCACCA |
|  | Navba_41335_PCR_F2 | CTTTGGAGGTATCAGTGGG |
|  | Navba_41335_PCR_R2 | CAGAAACCAAAGCATCACC |
|  | Navba_41335_3RACE_TD_P1 | GGGATTACGGGCAAAGCAGGTATACTC |
|  | Navba_41335_5RACE_TD_P1 | GGTGGATCAGTTGAGGGAAGTTGACAA |
