# Supplementary material for: Voltage-Gated Na+ Channel Isoforms and Their mRNA Expression Levels and Protein Abundance in Three Electric Organs and the Skeletal Muscle of the Electric Eel Electrophorus electricus
Source: PLoS One. 2016 Dec 1;11(12):e0167589. doi: 10.1371/journal.pone.0167589 (PMC5132174; doi:10.1371/journal.pone.0167589)
Supplement: S2 Table — (DOCX) [file pone.0167589.s007.docx]

**S2 Table.** Amino acid sequences or translated nucleotide sequences of Scn/SCN obtained from Genbank or UniProtKB/TrEMBL and their accession numbers used in classification tables and multiple sequence alignments.

| Species | Scn isoform | Accession number |
| --- | --- | --- |
| *Electrophorus electricus* | Scn4aa | KX575860 (*This study*) |
| *Sternopygus macrurus* | Scn4aa | AAK55442.2 |
| *Danio rerio* | Scn4aa | ABA54921.1 |
| *Takifugu rubripes* | Scn4aa | Q2XVR7.1 |
| *Tetraodon nigroviridis* | Scn4aa | ABB29443.2 |
| *Electrophorus electricus* | Scn4ab | KX575856 (*This study*) |
| *Sternopygus macrurus* | Scn4ab | AF378139.2 |
| *Danio rerio* | Scn4ab | ABA54920.1 |
| *Tetraodon nigroviridis* | Scn4ab | ABB29444.1 |
| *Takifugu rubripes* | Scn4ab | Q2XVR6.1 |
|  |  |  |
| *Electrophorus electricus* | Scn1b | KX575857 (*This study*) |
| *Sternopygus macrurus* | Scn1b | ABO30514.1 |
| *Danio rerio* | Scn1b | NP_001071007.1 |
| *Esox lucius* | Scn1b | XP_010903660.1 |
| *Larimichthys crocea* | Scn1b | XP_010743342.1 |
| *Oreochromis niloticus* | Scn1b isoform X2 | XP_005456107.1 |
| *Oreochromis niloticus* | Scn1b isoform X1 | XP_003451292.1 |
| *Haplochromis burtoni* | Scn1b | XP_005948306.1 |
| *Danio rerio* | Scn1bb | ABQ82131.1 |
| *Mus musculus* | SCN1B | AAH09652.1 |
| *Homo sapiens* | SCN1B | AAA61277.1 |
| *Rattus norvegicus* | SCN1B | AAH94523.1 |
| *Electrophorus electricus* | Scn2b | KX575858 (*This study*) |
| *Astyanax mexicanus* | Scn2b | XP_007239140.1 |
| *Danio rerio* | Scn2b | AAI62967.1 |
| *Haplochromis burtoni* | Scn2b | XP_005944721.1 |
| *Oreochromis niloticus* | Scn2b | XP_005474564.1 |
| *Larimichthys crocea* | Scn2b | XP_010750330.1 |
| *Esox lucius* | Scn2b | XP_010871694.1 |
| *Homo sapiens* | SCN2B | AAH36793.1 |
| *Mus musculus* | SCN2B | EDL25636.1 |
| *Rattus norvegicus* | SCN2B | AAC52967.1 |
| *Electrophorus electricus* | Scn4b | KX575859 (*This study*) |
| *Danio rerio* | Scn4ba | NP_001071038.1 |
| *Esox lucius* | Scn4b | XP_010871440.1 |
| *Haplochromis burtoni* | Scn4b | XP_005944722.1 |
| *Oreochromis niloticus* | Scn4b | XP_003441768.1 |
| *Danio rerio* | Scn4bb | AAI62294.1 |
| *Mus musculus* | SCN4B | AAU05394.1 |
| *Homo sapiens* | SCN4B | AAN74584.1 |
| *Xenopus (Silurana) tropicalis* | Scn4b | AAH88037.1 |
| *Rattus norvegicus* | SCN4B | NP_001008880.1 |
