# Supplementary material for: Voltage-Gated Na+ Channel Isoforms and Their mRNA Expression Levels and Protein Abundance in Three Electric Organs and the Skeletal Muscle of the Electric Eel Electrophorus electricus
Source: PLoS One. 2016 Dec 1;11(12):e0167589. doi: 10.1371/journal.pone.0167589 (PMC5132174; doi:10.1371/journal.pone.0167589)
Supplement: S3 Table — (DOCX) [file pone.0167589.s008.docx]

**S3 Table.** Primer sequences used for qPCR

| Gene | Primer Name | Primer sequence (5’ to 3’) |
| --- | --- | --- |
| *scn4aa* | Nav1.4a_1284_qPCR_F1 | AACCAAGAGGCAGAGATAACAG |
|  | Nav1.4a_1284_qPCR_R2 | AGTACTGGCAGCCTTATGCTT |
| *scn4ab* | Nav1.4b_1991+8400_qPCR_F2 | CAGACATGGACCAGCTAGAC |
|  | Nav1.4b_1991+8400_qPCR_R2 | GTCTAGCTGGTCCATGTCTG |
| *scn1b* | Navβ1a_31765+15676_qPCR_F2 | CTACTTTGACCGAACGCTC |
|  | Navβ1a_31765+15676_qPCR_R2 | CTGTTGGTGGAGATGGTC |
| *scn2b* | Navβ2_54714+33291_qPCR_F1 | ACCTGTCCATCACCATCTC |
|  | Navβ2_54714+33291_qPCR_R1 | GGTGAAATGCGTGCGTA |
| *scn4b* | Navβ4a_41335_qPCR_F2 | CTTGTTGAGAATGTGGACGA |
|  | Navβ4a_41335_qPCR_R2 | TCGCCCTCATCCTCAA |
