# Supplementary material for: Voltage-Gated Na+ Channel Isoforms and Their mRNA Expression Levels and Protein Abundance in Three Electric Organs and the Skeletal Muscle of the Electric Eel Electrophorus electricus
Source: PLoS One. 2016 Dec 1;11(12):e0167589. doi: 10.1371/journal.pone.0167589 (PMC5132174; doi:10.1371/journal.pone.0167589)
Supplement: S4 Table — (DOCX) [file pone.0167589.s009.docx]

**S4 Table.** Nucelotide coding sequences of *scn* obtained from Genbank or Ensembl and their accession numbers used for phylogenetic analysis.

| Species | Gene | Accession number |
| --- | --- | --- |
| *Danio rerio* | *scn1ba* | NM_001077539.3 |
| *Danio rerio* | *scn1bb* | EF394326.1 |
| *Electrophorus electricus* | *scn1b* | KX575857 (*This study*) |
| *Esox lucius* | *scn1b* | XM_010905358.1 |
| *Gadus morhua* | *scn1bb* | ENSGMOT00000014622.1 |
| *Gasterosteus aculeatus* | *scn1bb* | ENSGACT00000003292.1 |
| *Haplochromis burtoni* | *scn1b* | XM_005943554.2 |
| *Oreochromis niloticus* | *scn1b* | XM_005452811.2 |
| *Oryzias latipes* | *scn1b* | XM_011485848.1 |
| *Sternopygus macrurus* | *scn1b* | EF440447.1 |
|  |  |  |
| *Danio rerio* | *scn2b* | NM_001077629.1 |
| *Electrophorus electricus* | *scn2b* | KX575858 (*This study*) |
| *Esox lucius* | *scn2b* | XM_010873392.2 |
| *Gadus morhua* | *scn2b* | ENSGMOT00000007761.1 |
| *Gasterosteus aculeatus* | *scn2b* | ENSGACT00000009642.1 |
| *Haplochromis burtoni* | *scn2b* | XM_005944659.2 |
| *Oreochromis niloticus* | *scn2b* | XM_005474507.2 |
| *Oryzias latipes* | *scn2b* | XM_004075776.2 |
|  |  |  |
| *Danio rerio* | *scn4ba* | NM_001077570.1 |
| *Danio rerio* | *scn4bb* | NM_001077573.1 |
| *Electrophorus electricus* | *scn4b* | KX575859 (*This study*) |
| *Esox lucius* | *scn4b* | XM_010873138.1 |
| *Gadus morhua* | *scn4ba* | ENSGMOT00000017750.1 |
| *Gadus morhua* | *scn4bb* | ENSGMOT00000000040.1 |
| *Gasterosteus aculeatus* | *scn4ba* | ENSGACT00000009647.1 |
| *Gasterosteus aculeatus* | *scn4bb* | ENSGACT00000026964.1 |
| *Haplochromis burtoni* | *scn4b* | XM_005944660.2 |
| *Oreochromis niloticus* | *scn4ba* | ENSONIT00000007030.1 |
| *Oreochromis niloticus* | *scn4bb* | ENSONIT00000005523.1 |
| *Oryzias latipes* | *scn4ba* | ENSORLT00000015126.1 |
| *Oryzias latipes* | *scn4bb* | ENSORLT00000011255.1 |
|  |  |  |
| *Latimeria chalumnae* | *scn4A* | ENSLACT00000011066.1 |
